# Supplementary material for: Comorbid Hypertension Reduces the Risk of Ventricular Arrhythmia in Chronic Heart Failure Patients with Implantable Cardioverter-Defibrillators
Source: J Clin Med. 2022 May 17;11(10):2816. doi: 10.3390/jcm11102816 (PMC9146543; doi:10.3390/jcm11102816)
Supplement: Supplementary file 1 [file jcm-11-02816-s001.zip › jcm-1719643-supplementary.pdf]

Table S1: Baseline characteristics of patients at ICD implantation and balancing tests before and after inverse probability weighting and entropy balancing.

**Characteristics**	Hypertension		Non-hypertension			Standard mean difference		
	Before weighting	After IPTW	Before weighting	After IPTW	After EB	Before weighting	After IPTW	After EB
n	424		540					
Age	63.1	60.9	55.6	61.2	63.1	0.606	0.027	<0.001
Male (%)	80.7	81.1	77.8	79.6	80.7	0.071	0.038	<0.001
Body Mass Index	25.6	25.1	24.2	25.1	25.6	0.400	0.014	<0.001
Heart rate	68.5	68.8	67.0	68.7	68.5	0.037	0.010	<0.001
Systolic blood pressure	125.5	120.6	115.1	120.6	125.5	0.681	0.002	<0.001
Diastolic blood pressure	75.6	73.8	71.3	73.7	75.6	0.423	0.009	<0.001
Smoking	47.9	48.4	44.3	47.1	47.9	0.073	0.026	<0.001
Alcohol use	38.9	37.6	34.3	36.6	38.9	0.097	0.022	<0.001
SCD family history	5.0	4.5	4.8	4.5	5.0	0.006	<0.001	<0.001
Ablation history	7.1	6.3	9.8	6.9	7.1	0.099	0.025	<0.001
NYHA class						0.059	0.013	<0.001
I/II	62.5	59.5	59.1	60.1	62.5			
III/IV	37.5	40.5	40.4	39.9	37.5			
ICD primary prevention	33.7	37.0	33.0	36.0	33.7	0.016	0.020	<0.001
Syncope	40.1	42.1	47.5	41.2	40.1	0.129	0.018	<0.001
Frequent PVCs	45.3	43.9	43.1	45.2	45.3	0.043	0.025	<0.001
Atrial fibrillation	30.7	31.5	29.1	30.1	30.7	0.035	0.030	<0.001
Atrioventricular block	12.5	12.7	12.6	13.0	12.5	0.003	0.009	<0.001
Coronary arterial disease	61.8	53.1	36.3	53.3	61.8	0.527	0.006	<0.001
Stroke	9.0	7.1	4.4	6.7	9.0	0.181	0.015	<0.001
Pulmonary hypertension	8.5	9.3	8.5	8.8	8.5	0.001	0.017	<0.001

Diabetes mellitus	28.5	22.1	13.3	21.5	28.5	0.380	0.016	<0.001
Hyperlipidemia	68.2	57.0	35.2	57.1	68.2	0.699	0.004	<0.001
Hyperuricemia	13.0	10.3	8.3	10.7	13.0	0.151	0.012	<0.001
eGFR<60 ml/min/1.73m^2	32.8	25.7	16.7	25.3	32.8	0.380	0.008	<0.001
Antiarrhythmic drugs	58.3	58.0	61.7	57.4	58.3	0.070	0.012	<0.001
RAAS inhibitors	77.6	72.8	65.6	72.2	77.6	0.269	0.013	<0.001
β-blockers	91.3	92.1	90.4	92.2	91.3	0.031	0.003	<0.001
Calcium channel blockers	17.9	7.0	3.7	7.0	17.9	0.470	0.001	<0.001
Loop diuretics	70.5	75.8	73.5	74.3	70.5	0.067	0.035	<0.001
Digoxin	25.2	25.3	23.9	24.4	25.2	0.031	0.020	<0.001
Mineralcorticoid receptor antagonist	60.1	68.3	70.0	68.2	60.1	0.208	0.002	<0.001
Antiplatelets	43.9	38.9	28.1	39.5	43.9	0.332	0.013	<0.001
Anticoagulants	21.9	20.5	19.1	19.9	21.9	0.071	0.015	<0.001
Statin	64.2	57.2	42.8	57.1	64.2	0.439	0.002	<0.001
Left ventricular ejection fraction	42.8	41.3	40.9	41.5	42.8	0.145	0.020	<0.001
Left atrial diameter	44.2	44.0	43.1	43.6	44.2	0.143	0.046	<0.001
Left ventricular mass index	150.2	151.5	151.0	150.1	150.2	0.016	0.025	<0.001
Right ventricular diameter	23.18	23.1	23.5	23.0	23.18	0.058	0.021	<0.001
NT-proBNP	1707.9	1586.0	1475.6	1569.2	1707.9	0.105	0.008	<0.001
Hemoglobin	141.3	141.5	142.2	141.1	141.3	0.047	0.023	<0.001
LDH	206.1	205.4	205.3	204.6	206.1	0.011	0.010	<0.001
ESR	11.6	11.7	10.9	11.8	11.6	0.056	0.012	<0.001
TC	3.90	3.98	4.07	3.97	3.90	0.156	0.016	<0.001
HDL	1.09	1.12	1.14	1.12	1.09	0.104	0.011	<0.001

LDL	2.31	2.37	2.43	2.35	2.31	0.143	0.028	<0.001

RAAS inhibitors, Renin-angiotensin-aldosterone System inhibitors; other abbreviations as in Table 1.

Table S2. Associations between Hypertension diagnosis and the primary endpoint of VA using competing risk model in the crude analysis, multivariable analysis, propensity-score analysis and entropy-balanced analysis.

**Analysis**	**VA events in competing risk model HR (95% CI)**	**p value**
Crude analyses	0.67 (0.54-0.82)	<0.001
Multivariable analyses*	0.79 (0.63-0.98)	0.032
Propensity-score analyses		
With inverse probability weighting†	0.75 (0.57-0.97)	0.029
With matching‡	0.72 (0.54-0.95)	0.027
Adjusted for propensity score§	0.76 (0.60-0.97)	0.028
Entropy-balanced weighting analyses⁎	0.71 (0.51-1.00)	0.048

* Shown is the hazard ratio from the multivariable Fine-Gray sub-distribution hazard model, with additional adjustment for all demographic characteristics, comorbidities, echocardiographic parameters, medication, and laboratory parameters. Hypertension, together with age, sex, ICD prevention indication, coronary atrial disease, pulmonary hypertension, right ventricular diameter, and calcium channel blockers remained in the final model. The analysis included all 964 patients.

† Shown is the primary analysis with a hazard ratio from the multivariate Fine-Gray sub-distribution hazard model model adjusted with the same covariates with inverse probability weighting according to the propensity score. The analysis included all the patients.

‡ Shown is the hazard ratio from a multivariable Fine-Gray sub-distribution hazard model with the same covariates with matching according to the propensity score. The analysis included 482 patients (241 with hypertension and 241 without).

§ Shown is the hazard ratio from a multivariable Fine-Gray sub-distribution hazard model with additional adjustment for the propensity score. The analysis included all the patients.

⁎Shown is the hazard ratio from a multivariable Fine-Gray sub-distribution hazard model using weights from entropy balancing.The analysis included all the patients.

Figure S1. Love plot displaying absolute standardized differences comparing 46 baseline characteristics between chronic heart failure patients with or without comorbid hypertension before and after inverse probability weighting and entropy balancing.

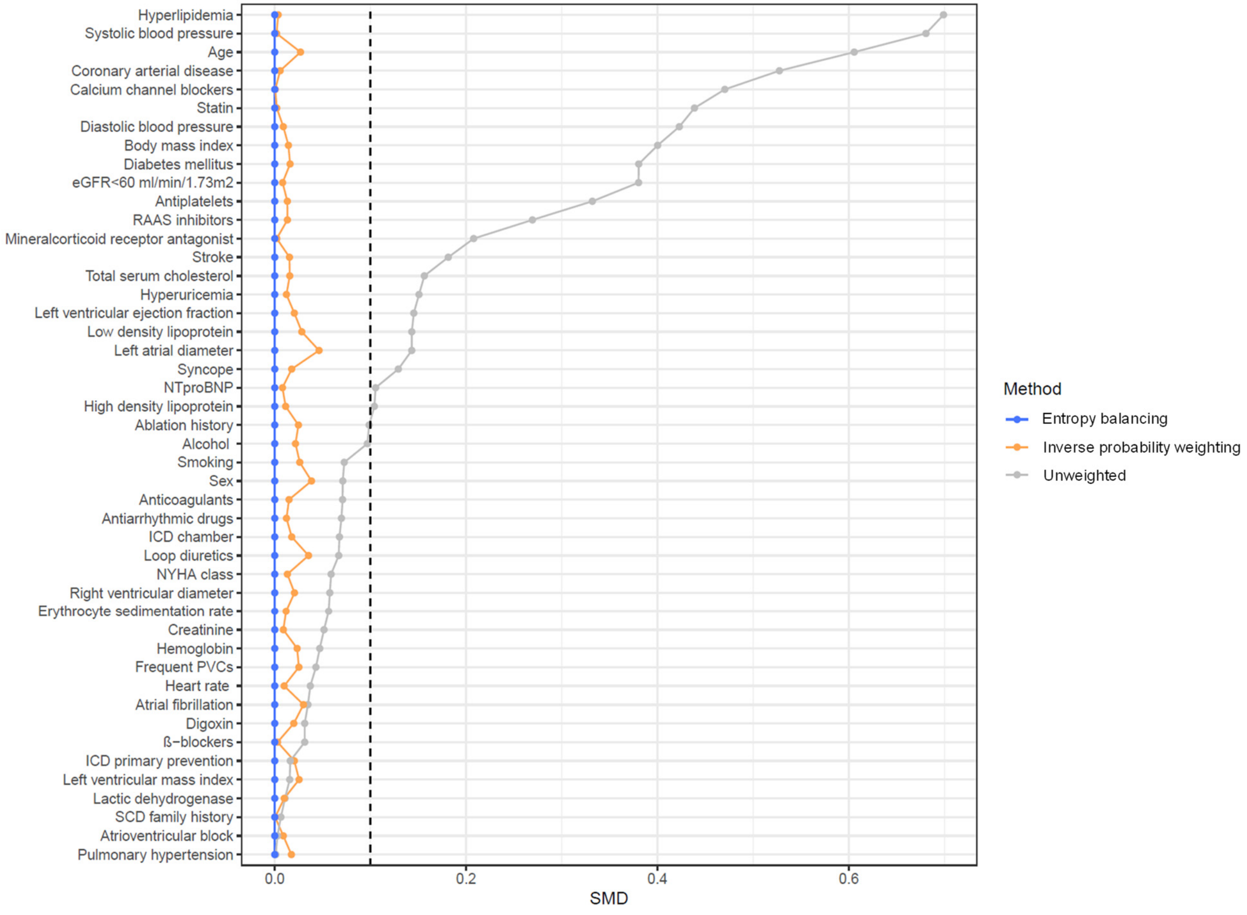

Figure S2. Entropy balancing weighted Kaplan–Meier time-to-event curves for the cumulative incidence of primary outcome events for patients with different (A) grades and (B) controlled status of hypertension.

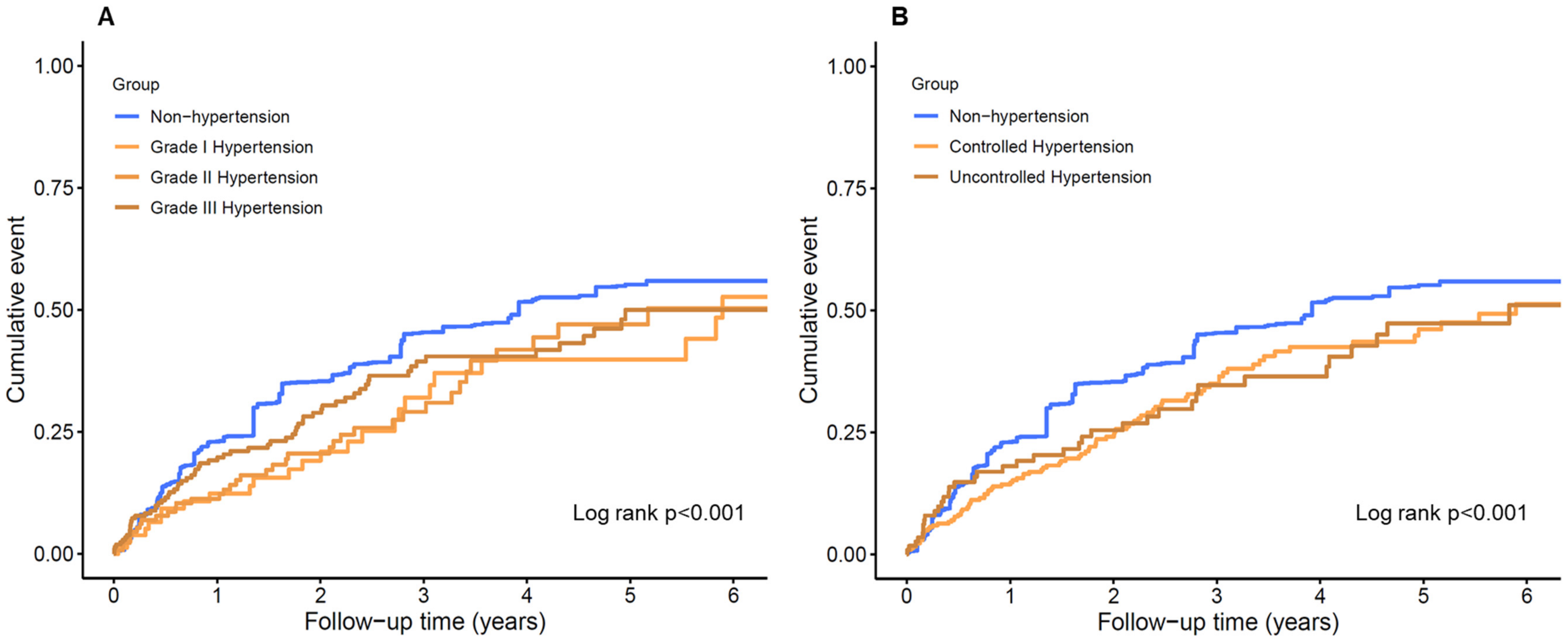

Figure S3. Restricted Cubic Spline Plots for Primary Outcome by Diastolic Blood Pressure.

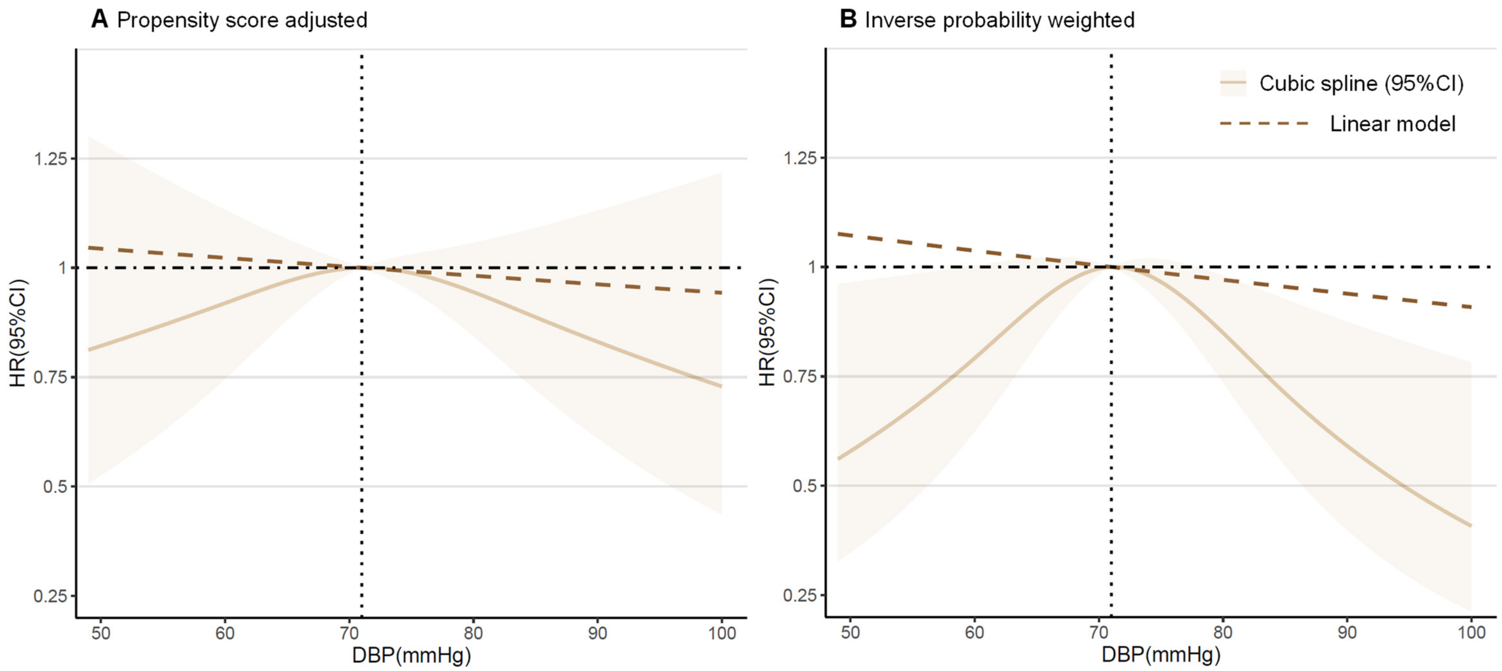

Hazard ratios and 95% confidence intervals for primary VA outcome by diastolic blood pressure level in 964 patients with chronic heart failure according to restricted cubic spline regression models using 3 knots. Solid black lines indicate hazard ratios, and shaded areas indicate 95% CI. Plots on the left panel (A) are adjusted for propensity scores, and those on the right panel (B) are inverse probability weighted on 46 baseline characteristics.
